# Effects of a 24-Week Exercise Program on Functional Fitness, Oxidative Stress, and Salivary Cortisol Levels in Elderly Subjects

**DOI:** 10.3390/medicina58101341

**Published:** 2022-09-23

**Authors:** Gabriele Morucci, Larisa Ryskalin, Simone Pratesi, Jacopo J. V. Branca, Alessandra Modesti, Pietro Amedeo Modesti, Massimo Gulisano, Marco Gesi

**Affiliations:** 1 Department of Translational Research and New Technologies in Medicine and Surgery, University of Pisa, 56126 Pisa, Italy; 2 Center for Rehabilitative Medicine “Sport and Anatomy”, University of Pisa, 56121 Pisa, Italy; 3 Department of Experimental and Clinical Medicine, University of Florence, 50134 Florence, Italy; 4 Department of Biomedical, Experimental and Clinical Sciences “Mario Serio”, University of Florence, 50134 Florence, Italy; 5 Sport Medicine Unit, Careggi University Hospital, University of Florence, 50134 Florence, Italy

**Keywords:** physical activity, exercise program, training, functional fitness, physical fitness, elderly people, health promotion

## Abstract

*Background and Objectives:* Aging is a biological and irreversible process characterized by physiological alterations resulting in a progressive decline in biological functions, decreased resistance or adaptability to stress, and increased disease susceptibility. A decline in functional fitness, imbalance between pro- and antioxidant capacity, and/or hormonal dysregulation adversely impact physical capacity, emotional status, and overall quality of life, especially within the elderly population. On the other hand, regular physical activity is considered an effective strategy to prevent and reduce those changes associated with primary aging and concurrent chronic disease, while slowing age-related physical degeneration. However, there is still limited evidence-based information regarding both the intensity and interval of effective interventions on physical functioning in older adults. Thus, the aim of the study was to assess the effects of a 24-week regular multimodal exercise program on functional fitness, oxidative stress, salivary cortisol level, and self-perceived quality of life in a group of eighteen physically active elderly subjects (mean age 72.8 ± 7.5 years). *Materials and Methods:* A set of anthropometric and physical measurements (grip strength, chair sit to stand, sit and reach and back scratch) assessing the functional fitness performance were evaluated. Moreover, biochemical markers (derived-reactive oxygen metabolites (d-ROMs) and the biological antioxidant potential (BAP) tests, and salivary cortisol levels) and the EuroQoL 5-Dimension 3-Level (EuroQoL 5-D 3-L) self-perceived questionnaire of quality of life were measured before and after the intervention program. All measurements were normally distributed as assessed by D’Agostino and Pearson’s omnibus normality test. Student’s *t*-tests were used to evaluate the differences in all the parameters measured at baseline (T0) and after the 24-week physical program (T1). *Results:* The results showed that an age-tailored structured intervention exercise program (1 h per session, twice per week, for 24 weeks) was effective in improving flexibility and other biomechanical parameters, such as muscle strength and the dynamic balance fitness component, which are key to performing daily tasks independently. Moreover, biochemical analyses demonstrate that the proposed intervention program has beneficial effects on the balance between plasma ROS production and their neutralization. *Conclusions:* The results confirm the benefits of regular physical activity in older adults resulting in improved physical strength and flexibility in the functional fitness parameters, and in regulating anti- and pro-oxidant activity and cortisol (stress hormone) levels.

## 1. Introduction

Aging is a biological and irreversible process characterized by physiological alterations that result in a progressive decline in overall biological and metabolic functions, decreased physical performance and resistance to stress, and increased disease susceptibility [1,2,3]. Age-related structural and functional changes affect a broad range of cells, tissues, and organs of the whole body [4]. This results in a reduction in musculoskeletal and joint mobility (affecting muscle strength, balance, and flexibility), a decline in cardiovascular and pulmonary functions (acting on blood pressure, gas exchange, and respiratory capabilities), as well as altering body composition and metabolism, influencing hormone levels and antioxidant defenses. This, in turn, negatively affects the physical capacity, psycho-emotional status, and global well-being of the elderly people. At the same time, functional limitations adversely impact the individual capacity to complete daily activities, and thus independent living [5].

Contrariwise, an active lifestyle supported by appropriate and regular exercise represents an effective strategy for disease prevention and health promotion, especially among the elderly population. Increasing evidence demonstrates that physical activity or increased mobility improves body organic conditions and maintains functional capacity while slowing age-related physical degeneration [6,7,8,9].

Despite numerous studies emphasizing the beneficial effects of regular physical activity on both psychophysical health and quality of life (QoL), older adults tend to be less active with advancing age, and only 22% of adults ≥ 65 years meet the recommendations for physical activity [10]. Moreover, some studies pointed out that functional adaptations and benefits induced by regular physical activity can be undone even after a short sedentary or detraining period, especially in elderly people [11].

Within aging populations, a major concern relates to the development of effective physical exercises aimed at (i) counteracting age-associated physical limitations and disabilities, such as a progressive decline in muscle strength and power; (ii) improving functional fitness performance and evaluating progress; and (iii) preventing future mobility issues. In this regard, “functional fitness” is defined as “the physiologic capacity to perform normal everyday activities safely and independently without undue fatigue” [12]. This includes components such as lower and upper limb strength, lower and upper limb flexibility, aerobic endurance, and dynamic balance [12]. Within this frame, a fitness test battery especially for older adults was developed and validated by Rikli and Jones [13], pointing out that being able to perform daily tasks requires the ability to perform functional movements that strictly depend on having a sufficient level of physiologic reserves such as strength, flexibility, balance, agility, and endurance [14].

Although different types of exercise intervention have been claimed to be effective in improving physical health in older adults, at present, there is no clear consensus regarding the most effective treatment to help these subjects remain robust and independent. In fact, there is conflicting and/or fragmented evidence concerning which exercise characteristics (e.g., intensity, interval, frequency, type of exercises) are most likely to be effective in improving physical functioning in older adults, making the comparisons among different studies highly challenging [15,16,17,18]. On the other hand, within the elderly population, and especially in community settings, several factors (e.g., age, physical fitness level, psychological health, onset of chronic diseases, hospital admission) can lead to training cessation and thus physically inactive periods [19]. These, in turn, may neutralize the beneficial effects of age-tailored training programs aimed at slowing physical degeneration related to aging.

Taking the aforementioned into account, we can hypothesize that the detraining influence on functional fitness parameters, as well as on antioxidant biomarkers, seems to be closely related to its length; thus, the longer the period of detraining, the worsening of these parameters. Thus, there is still a need for further studies aimed at investigating whether even a short period of detraining may negatively affect all or some of the components that characterize functional fitness in physically active elderly subjects routinely participating in senior gym courses.

Furthermore, accurate and objective monitoring and evaluation of individuals’ physical adaptability to activity programs is key to maximizing the beneficial effects of physical activity itself as well as reducing possible adverse events or injuries.

Therefore, the aim of the present study is to assess the effectiveness of a 24-week regular multimodal exercise program based on functional fitness exercises, and focused on the improvement of body flexibility, in a group of 18 physically active elderly people (>60 years old) after a short sedentary/detraining period.

## 2. Materials and Methods

### 2.1. Design and Participants

A total of eighteen elderly subjects, aged between 62 and 86 years (mean age 72.8 ± 7.5 years), was recruited from a cohort of physically active practitioners participating in a standard senior fitness class in a gym center (Olimpia Gym, Danilo Innocenti Multipurpose Sports Center), located in Florence, Italy. Of the eighteen study participants, fourteen were women (72.9 ± 7.4 years), and four were men (72.6 ± 9.1 years).

Regarding the inclusion criteria, all the participants had to be aged over 60 years, presenting similar socio-demographic status, physically independent, non-smoking, non-alcoholic, non-diabetic, and have no other health conditions that could potentially compromise the capability to perform the activity program and the physical tests. 

Participants under medications (mostly anti-hypertensives and anti-coagulants) were required not to alter their medical treatments during the intervention program. Contrariwise, people under hormone replacement, anti-resorptive, or corticosteroid therapies were considered not eligible for inclusion in this study.

In addition, in order to limit potential confounding factors, all the subjects were instructed not to participate in any other moderate-intensity physical activities and not to alter dietary and lifestyle habits during the entire intervention program. 

Before the beginning of the study, all the procedures were fully explained, and written informed consent was provided by all the participants prior to enrolment in the study, which was conducted according to the policy statement set forth in the Declaration of Helsinki. All the experiments were conducted according to established ethical guidelines. The study was approved by the local ethics committee of the University of Florence, Italy (AM Gsport 15840/CAM_BIO, date of approval 24 February 2020).

To investigate the beneficial effects of a 24-week physical activity intervention based on functional fitness exercises in an older adult population, all participants were investigated at baseline (T0) and at the end (T1) of the exercise program. Data collection consisted of (i) anthropometric measurements including body weight, body fat percentage, and body mass index (BMI); (ii) functional fitness tests regarding strength, flexibility, aerobic endurance, and dynamic balance; and (iii) biochemical tests to assess oxidative stress and salivary cortisol levels. In addition, a self-perceived QoL questionnaire (EuroQoL 5-D 3-L) was also used to have a subjective evaluation of the indirect effects of the physical activity program intervention on the overall health-related QoL.

### 2.2. Physical Exercise Intervention Program

The physical exercise intervention program was carried out for 24 weeks, from January 2017 to June 2017, and held twice a week (on Monday and Thursday), with each session lasting approximately 60 min. The physical intervention consisted of a multimodal exercise program based on functional fitness exercises, for both the upper and lower body, and mostly focused on the flexibility component.

The cardiorespiratory warm-up consisted of 10 min of indoor/outdoor walking increasing from 2.5 to 3.5 km/h.

The specific fitness exercise section included 35–40 min of muscular tasks for coordination/balance and flexibility training in which all joints and major muscle groups were exercised in an increased number of repetitions per session. Muscle groups targeted in the lower limbs included hip flexors/extensors and abductors/adductors, knee flexors and extensors, ankle plantar flexors and dorsiflexors; in the upper extremity they included shoulder flexors/extensors and abductors, and elbow flexors/extensors; in the trunk region, they involved neck and trunk flexors/extensors. Coordination and balance exercises were undertaken using equipment such as chairs, elastic bands, and sticks. The final cool-down component of each session consisted of 10 min of gentle stretching exercises targeting muscle groups.

Exercises were undertaken in group activities with a major emphasis on social interaction and enjoyment. All study participants completed the training protocol. Each participant’s attendance was recorded for every training session. The mean attendance to the intervention program was >80% (at least 39 of 48 sessions). During and after each training session or physical test, none of the participants suffered injuries because of the exercise program or physical tests. No adverse events occurred during the 24-week intervention.

The exercise program intervention was conducted by a physical instructor with more than 10 years of experience in senior fitness.

### 2.3. Anthropometric Measurements

Sex and age of birth of each participant were recorded at baseline (T0). 

Anthropometric measurements (body weight, body fat percentage, BMI) were carried out before the beginning (baseline, T0) and after 24 weeks of the training intervention program (T1).

Body weight and height were measured to the nearest 0.1 kg (Seca 761, Seca Weighing and Measuring Systems, Birmingham, UK) and 0.1 cm (Seca 220), respectively.

A body fat monitor (OMRON BF306, OMRON Healthcare, Hoofddorp, The Netherlands) was used to measure the body fat percentage by a bioimpedance analysis method.

Body mass index (BMI) was calculated using the standard formula weight/height^2^ (kg/m^2^).

### 2.4. Functional Fitness Tests

**Figure 1:**
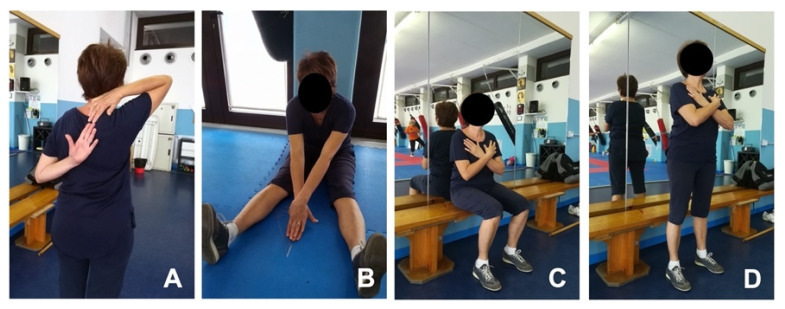
Examples of some senior functional fitness tests (FFTs) used in the present study. (**A**) BST; (**B**) V-SRT; (**C**,**D**) sitting position and full stand in CSST, respectively.

Senior functional fitness tests (FFTs) (Figure 1) were performed at T0 and T1 in two dedicated extra sessions, after a standard warm-up of 10 min.

Data were obtained from different tests included in a usual senior FFTs battery [20] encompassing:

− handgrip strength test (HST): upper body strength;
− chair sit-to-stand test (CSST): lower body strength;
− back scratch test (BST): upper body (shoulder) flexibility;
− v-sit and reach test (V-SRT): lower body (lower back and hamstring muscles) flexibility;
− timed up and go test (TUG): dynamic balance
− six-min walk test (6MWT): aerobic endurance.

All the above tests were performed on the same day and in the same order for each study participant, under the supervision of a physical instructor. 

#### 2.4.1. Handgrip Strength Test (HST) 

HST was performed with the dominant hand by an electronic hand dynamometer to the nearest 0.1 kg (Camry EH101, Zhongshan Camry Electronic Co., Zhongshan, Guangdong Province, China). From a sitting position, with the elbow flexed at 90 degrees and the forearm semi-pronated, the subjects were asked to squeeze the dynamometer as hard as possible for 10 s. The procedure was repeated three times with 1 min resting period between each squeeze to overcome the fatigue. The best peak grip value among the three squeezes was taken as a score [21,22].

#### 2.4.2. Chair Sit-to-Stand Test (CSST)

The score represents the number of full stands completed in 30 s from a sitting position on a standard chair without armrests (seat height approx. 45 cm) and with the arms folded across the chest [23].

#### 2.4.3. Back Scratch Test (BST)

From a standing position, the subject was instructed to bring the hands towards the back, with one hand from above the shoulder and the contralateral hand from the middle of the back (hand palms facing each other). The measurement represents the distance to the nearest half-centimeter between the tips of the extended middle fingers. Values were recorded as negative if there was a distance between the fingers or positive in case of overlapping. The test was performed twice, and the best score was retained. The procedure was reversed, and the assessment of the opposite shoulder was also performed [14]. As demonstrated by Rikli and Jones [12] the BST is a reliable and valid measure of overall shoulder range of motion (i.e., shoulder joint and arch flexibility in older adults).

#### 2.4.4. V-Sit and Reach Test (V-SRT)

During the V-SRT test, the subject sat on the floor with the feet approximately 30 cm apart forming a V-shaped leg position (knees extended, ankles dorsiflexed, and back pressed against the wall). From this starting position, the subject was asked to slowly stretch out forward as far as possible with the hands placed on top of each other, palms facing downward, without bending the knees, elbows, or back. During the task, legs remained extended on the floor or kept pressed to the floor by the instructor, if necessary. The score represents the maximal reach distance measured from the starting position. The test was performed twice, and the best score was retained [24].

#### 2.4.5. Timed Up and Go Test (TUG)

Each subject started the test from a sitting position on a standard chair without armrests (seat height approx. 45 cm). Upon the start command, the subject was asked to get up from the seated position, walk a distance of 3 m (at a comfortable speed) in a straight line, and return to the seated position on the chair. The test was performed indoors on rubber flooring, with each subject wearing personal footwear. The time (in seconds) was measured from the moment the subject stood up from the chair until the subject’s back was positioned against the back of the chair, after sitting down [25]. Time was measured with a stopwatch to the nearest 0.1 s. The test was performed twice, and the best score was retained.

#### 2.4.6. Six-Min Walk Test (6MWT)

The test measures the maximum distance (in meters) the participant is able to walk in 6 min. The test was performed twice with a 30 min resting period between each task, and the best score was retained. No subject requested a rest period during the tests. 

The test was performed outdoors on a running track of the multi-purpose sports center adjoining the indoor gym. All the participants were tested on the same day under similar weather conditions, and all the precautions and recommendations suggested for the execution of the test were followed according to the American Thoracic Society guidelines for the 6MWT [26].

### 2.5. Biochemical Analyses

#### 2.5.1. Stress Hormone

Saliva samples were collected from all participants, as described in Pacini et al. 2014 [27], at T0 and T1, by using a Salivette^®^ saliva collection device (Sarsted, Verona, Italy). Saliva samples were taken in the morning, at least 30 min after eating, drinking, and brushing the teeth to prevent any contamination. The participants were instructed to gently chew the swab in the mouth for at least 1 min until it was completely saturated with saliva. Saliva samples were centrifuged for 2 min at 1000× *g* and stored at −20 °C prior to analysis. Cortisol concentrations in the centrifugates were determined in duplicate using a competitive enzyme-linked immunosorbent colorimetric assay for quantitative determination according to the manufacturer’s instructions (Cortisol Saliva ELISA Kit, Grifols Italia, Pisa, Italy). The absorbance, inversely proportional to the cortisol concentration within the sample, was measured at 450 nm with a microplate photometer (Multiskan FC, Thermo Scientific, Milan, Italy).

#### 2.5.2. Oxidative Stress

The levels of derivatives (hydroperoxides) of reactive oxygen metabolites (derived-reactive oxygen metabolites (d-ROMs)) and the biological antioxidant potential (BAP) were measured at T0 and T1 by a scientifically certified oxidative stress integrated analytical system (FREE Carpe Diem, Diacron International, Grosseto, Italy) including a spectrophotometer, a mini centrifuge, and appropriate measurement kits. The d-ROMs test measures the plasma level of hydroperoxides (ROOH), such as hydrogen peroxide (H_2_O_2_), which reflects the amount of reactive oxygen species (ROS) from which they are formed. The BAP test provides an estimate of the overall antioxidant capacity by measuring the concentration of plasma antioxidants, which are able to reduce ferric iron to the ferrous state. Briefly, a blood sample from the fingertip capillary was collected from each subject in heparinized microvette tubes (Sarsted, Verona, Italy). Plasma was extracted by centrifugation (6000 rpm for 90 s at 4 °C) and stored at 4 °C prior to analysis.

For the d-ROMs test, 20 µL of plasma was added into a cuvette containing a pH 4.8 buffer and mixed by inversion to allow Fe^2+^ and Fe^3+^ separation from the plasma proteins; this, in turn, was expected to lead to decomposition of plasma hydroperoxides into alkoxyl and peroxyl radicals catalyzed by Fe^2+^ and Fe^3+^. Then, 20 µL of a chromogen (N,N-diethyl-paraphenylenediamine) was added to the solution to allow chromogen oxidation by free radicals yielding red-colored radical cations. The solution was placed in a spectrophotometer (Free Duo, Diacron International s.r.l, Grosseto, Italy), and radical cations were measured at 505 nm. Data were expressed in arbitrary units, Carratelli units (U.CARR), where 1 U.CARR corresponded to 0.08 mg/dL of H_2_O_2_ [28,29,30] (Table 1).

For the BAP test, 50 µL of a chromogenic reagent containing Fe^3+^ was added into a cuvette and mixed by inversion; the resulting red solution was measured by a spectrophotometer. Then, 10 µL of plasma was added to the cuvette and mixed. Thereafter, the solution was incubated for 5 min to allow the reaction of deoxidation. The intensity of the decoloration was detected with a spectrophotometer at 505 nm. Data were expressed in µmol/L of reduced ferric ions [30,31,32] (Table 2).

### 2.6. QoL Questionnaire

At the beginning of the study and after 24 weeks of physical intervention, the self-perceived QoL questionnaire EuroQoL 5-Dimension 3-Level (EuroQoL 5-D 3-L) was applied to all participants in-person to evaluate their subjective perception of the indirect effects of the physical activity program intervention on the overall well-being and generic QoL. The EuroQoL 5-D 3-L is a preference-based, self-reported description of the subject current health in five dimensions that include mobility, self-care, usual activities, pain/discomfort, and anxiety/depression, each one having three levels of severity (i.e., severe, moderate, or none). The questionnaire also includes a visual analogue scale (EQ-VAS) recording the participant’s self-rated health status on a vertical scale with endpoints labelled “best imaginable health” (i.e., 100) and “worst imaginable health” (i.e., 0) [33]. This questionnaire was selected because it is validated, widely used, and translated into the Italian language.

### 2.7. Statistical Analysis

The normality of the distributions for each variable was assessed using D’Agostino and Pearson’s omnibus normality test (95% CI). All data showed a normal distribution, and Student’s *t*-tests were used to evaluate the differences in all the parameters measured at baseline (T0) and after the 24-week physical program (T1). Results were considered significant at *p* < 0.05 and highly significant at *p* < 0.01. Statistical analyses were performed using Statistical Analysis Systems (SAS/STAT module version 9.2; SAS Institute, Cary, NC, USA). Data are given as the mean ± SD.

## 3. Results

### 3.1. Assessment of Anthropometric Parameters

The demographic and anthropometric characteristics of all the subjects enrolled in the present study are shown in Table 3. 

At baseline, the mean BMI was 25.8 ± 2.0 kg/m^2^, indicating that our sample of patients fell within the normal or healthy weight range [34]. Only one subject out of 18 (5.5%) exceeded the 85th percentile of both weight and BMI range charts [35], thus resulting at risk of being overweight. Similarly, one study participant (5.5%) fell below the 5th percentile of the weight chart at the end of the training program. However, no statistically significant difference occurred in the mean weight and BMI following the 24-week exercise program.

At baseline, the mean body fat percentage was 37.2 ± 5.7. Only two subjects (11%) fell out of the normal range of the body fat chart [36]. It is noteworthy that after the intervention program, there was a slight, though significant, decrease in body fat percentage (Δ = −1.5% ± 3, *p* = 0.047) (Baseline 95% CI, 34.58 to 39.82; Post-intervention, 95% CI, 32.86 to 38.52).

### 3.2. Effects of 24-Week Intervention Program on Senior Functional Fitness Tests

#### 3.2.1. Upper and Lower Body Strength

**Figure 2:**
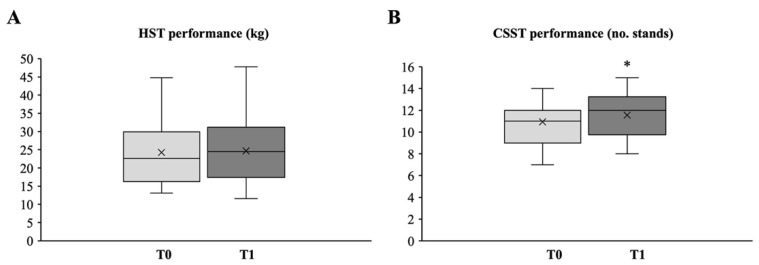
Box plot and whiskers diagram of the mean strength values for upper (**A**) and lower (**B**) body. The central line of the box represents the median, the top and bottom margins of the box represent the 1st and 3rd quartiles, respectively, and the ends of the whiskers represent the minimum and the maximum values. Mean values are presented with x. Statistically significant differences are indicated with * *p* < 0.05.

The differences in upper limb muscle strength measured by the HST between T0 and T1 were not significant (Figure 2A). However, an increasing trend (Δ = 0.42 ± 4.67 kg) was observed at the end of the exercise program. Noteworthily, all HST values both at T0 and T1 exceeded the 20% of relative body weight, i.e., the threshold suggested as necessary to perform daily tasks that require a firm grip (e.g., raising body weight onto a raised bus platform) [37]. 

On the other hand, a significant, albeit slight, increase in lower limb muscle strength was observed after the intervention (Figure 2B). The mean score of full stands assessed by CSST increased by 0.61±1.09, rising from 10.94 ± 1.86 (95% CI, 10.04 to 11.84) repetitions at T0 to 11.56 ± 2.12 (95% CI, 10.56 to 12.56) at T1 (*p* = 0.03). It should be noted that at T0, four subjects scored below the normal CSST range for age and sex, falling below the 25th percentile [23]. At the end of the training intervention program, an improvement in the performance was observed in one of them, who accomplished an age- and sex-matched CSST value between the 25th and 75th percentile.

#### 3.2.2. Upper and Lower Body Flexibility

**Figure 3:**
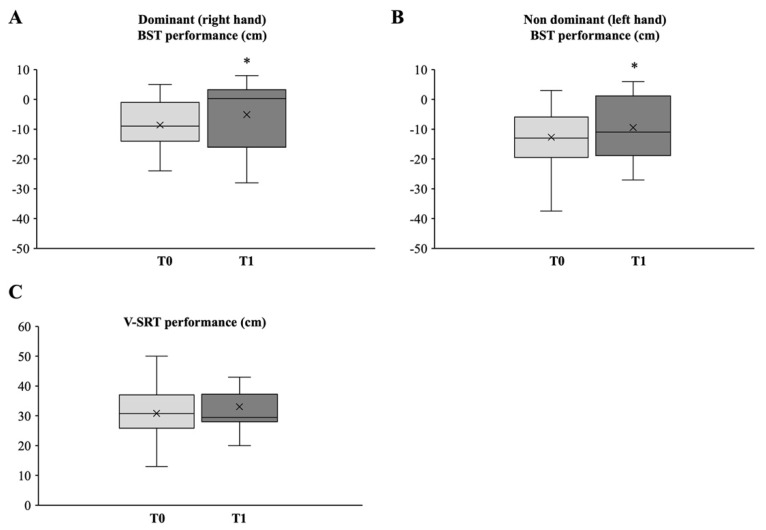
Box plot and whiskers diagram of the mean flexibility values for upper (**A**,**B**) and lower (**C**) body. The central line of the box represents the median, the top and bottom margins of the box represent the 1st and 3rd quartiles, respectively, and the ends of the whiskers represent the minimum and the maximum values. Mean values are presented with x. Statistically significant differences are indicated with * *p* < 0.05.

A significant increase in the upper body (shoulder) flexibility and overall shoulder range of motion (i.e., shoulder joint and arch flexibility) was observed after the 24-week exercise program as assessed by the BST test (Figure 3A,B). Both right (Figure 3A) and left (Figure 3B) glenohumeral joint flexibility showed an increment from −8.6 cm ± 8.2 (95% CI, −12.37 to −4.79) to −5.1 cm ± 10.2 (95% CI, −9.84 to 0.38) (Δ = 3.5 cm ± 5.3; *p* = 0.01) (Cohen’s d_s_ −0.37) and from −12.7 cm ± 10.3 (95% CI, −17.45 to −7.93) to −9.4 cm ± 10.4 (95% CI, −14.25 to −4.63) (Cohen’s d_s_ −0.31) (Δ = 3.3 cm ± 4.8; *p* = 0.01), respectively.

It is noteworthy that at baseline, 33% of the right and 56% of the left shoulder joint flexibility assessments did not fall within the BST average distance chart [23], turning into 22% and 44%, respectively, after the exercise program. 

Interestingly, at baseline, 3 out of 18 (16.5%) subjects showed results above average for the right shoulder joint test, being 4 out of 18 (22%) after the program. No above-average results were observed for the left shoulder joint. In this regard, it should be noted that the right arm was the dominant arm for all participants in this study.

In contrast, no statistically significant difference was observed in the lower body (hamstring and lower back) flexibility after the 24-week exercise program as assessed by the V sit-and-reach test (Figure 3C).

However, it is interesting to observe that according to the percentile ranks for the V sit-and-reach test [38], 3 out of 18 (16.5%) subjects at baseline showed results under the 20th percentile, and only 1 out of 18 (5.5%) after the exercise period, Moreover, 2 out of 18 (11%) showed results above the 80th percentile, both at baseline and after the training period.

#### 3.2.3. Dynamic Balance

**Figure 4:**
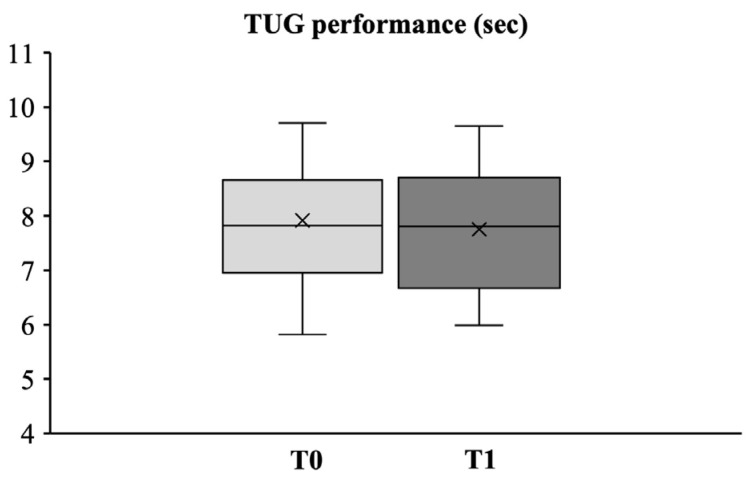
Box plot and whiskers diagram of the TUG assessing dynamic balance. The central line of the box represents the median, the top and bottom margins of the box represent the 1st and 3rd quartiles, respectively, and the ends of the whiskers represent the minimum and the maximum values. Mean values are presented with x.

As reported in Figure 4, TUG test evaluations assessed before and after the intervention program showed no statistically significant differences in the dynamic balance component.

#### 3.2.4. Aerobic Endurance

**Figure 5:**
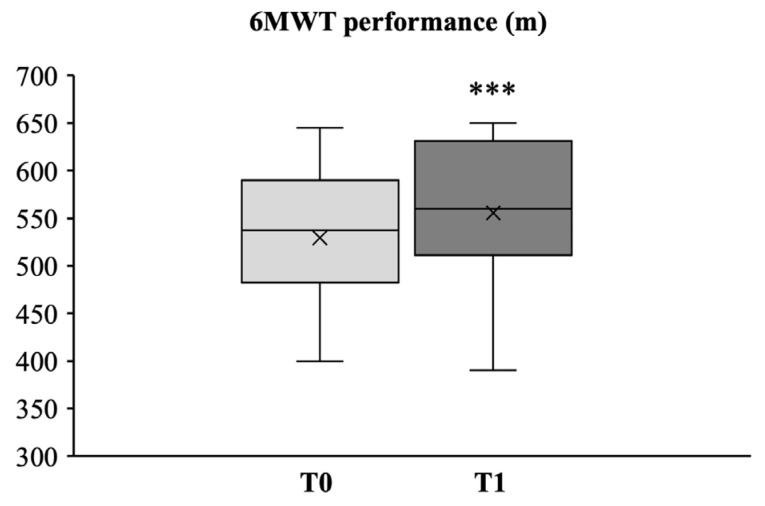
Box plot and whiskers diagram of the 6MWT assessing aerobic endurance. The central line of the box represents the median, the top and bottom margins of the box represent the 1st and 3rd quartiles, respectively, and the ends of the whiskers represent the minimum and the maximum values. Mean values are presented with x. Statistically significant differences are indicated with *** *p* < 0.001.

As reported in Figure 5, after the intervention program, the results of the six-min walk test (6MWT) showed a significant increase in the mean walked distance from 529.44 ± 73.46 mt (95% CI, 495.5 to 563.38) to 555.56 ± 80.93 mt (95% CI, 518.17 to 592.95) (Cohen’s d_s_ −0.33) (Δ = 26.11 ± 15.68 mt; *p* < 0.001). 

### 3.3. Effects of Functional Fitness on Salivary Cortisol and Plasma Oxidative Stress Levels

#### 3.3.1. Stress Hormone

**Figure 6:**
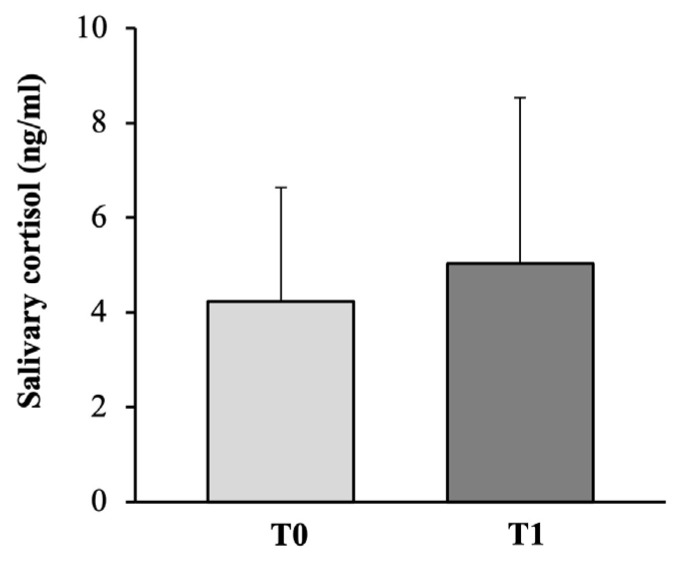
Graph reporting salivary cortisol levels at baseline and the end of the training program.

The results reported in Figure 6 revealed no significant differences between the salivary cortisol levels measured before and after the 24-week exercise program. Indeed, all the subjects fell within the normal reference for morning salivary cortisol concentrations (3–10 ng/mL), except for only one subject out of 18 (5.5%), who exceeded these reference values both at T0 (10.4 ng/mL) and T1 (15.5 ng/mL). 

#### 3.3.2. Oxidative Stress

The global assessment of oxidative stress on whole blood was performed by coupling d-ROM test (oxidant status assessment) with the BAP test (antioxidant power assessment).

**Figure 7:**
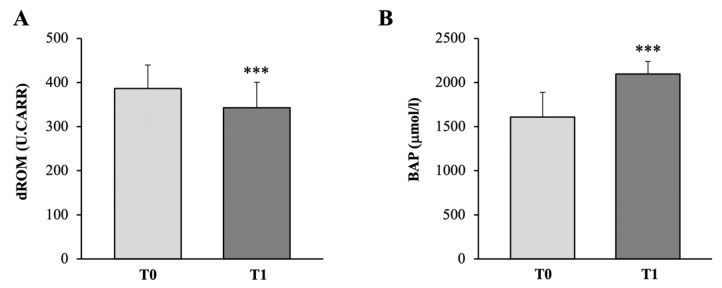
Graph reporting the plasma levels of d-ROM (**A**) and BAP (**B**), respectively. Statistically significant differences are indicated with *** *p* < 0.001.

As reported in the graph of Figure 7A, there was a significant decrease of 12% (*p* < 0.001) in d-ROM levels after 24-week physical activity program (339.80 ± 54.82 U.CARR) (95% CI, 314.48 to 365.12), compared to baseline values (386.73 ± 53.02 U.CARR) (95% CI, 362.24 to 411.22) (Cohen’s d_s_ 0.87). 

Along with this, a significant increase of 30% (*p* < 0.001) was observed in BAP average levels, increasing from 1607.84 ± 282.39 µmol/L (95% CI, 1477.39 to 1738.29) at T0 up to 2097.09 ± 144.31 µmol/L (95% CI, 2030.43 to 2163.75) at T1 (Cohen’s d_s_ −2.18) (Figure 7B).

### 3.4. Quality of Life Assessment

**Figure 8:**
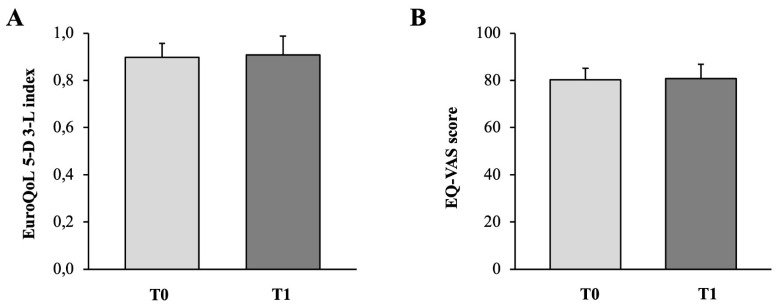
Graph reporting the EuroQoL 5-D 3-L index values (**A**) and the corresponding EQ-VAS scores (**B**), respectively.

The results of the EuroQoL 5-D 3-L health questionnaire may be converted from a descriptive system into a single index value [33]. As reported in the graph of Figure 8A, EuroQoL 5-D 3-L mean index values calculated before and after the exercise program showed no significant differences (0.90 ± 0.06 vs. 0.91 ± 0.08, respectively).

Along with this, also the corresponding EQ-VAS health scores provided no evidence for significant effects after the 24-week exercise program as emerged by the self-rated mean values at T0 and T1 (80.28 ± 4.85 vs. 80.83 ± 6.07, respectively) (Figure 8B).

## 4. Discussion

The present study was designed to evaluate the effects of a 24-week regular physical activity program focused on functional fitness-related exercises in 18 physically active older adult practitioners (aged between 62 and 86 years). In particular, the physical intervention consisted of a multimodal exercise program based on functional fitness exercises, for both the upper and lower body, and mostly focused on the flexibility component. Within the aging population, the maintenance of the fitness capacity needed to perform common activities of daily activities represents the most relevant aspect to preserve self-care, independence, and overall QoL [39,40,41,42,43].

In our study, as expected, we observed that after 24 weeks of functional fitness intervention, participants’ body weight and BMI remained unchanged because our program exercise was meant to induce neither a weight loss nor a reduction in BMI index. Although the physical exercise intervention program did not alter the mean weight and BMI, it was effective in decreasing body fat percentage, which can be considered an indicator of geriatric syndrome and sarcopenic obesity within elderly populations.

As demonstrated by this study, an age-appropriate structured intervention exercise program can improve flexibility and other biomechanical and physiological parameters, such as muscle strength and the aerobic endurance fitness component, which are key to performing daily tasks independently (i.e., climbing stairs, dressing, and eating independently) [44,45]. This is in line with previous studies demonstrating that a 1 h intervention program, including at least 10 min of warm-up, 30–40 min of main exercise, and 10–15 min of cool-down stages, 2/3 sessions per week, and continuing for at least 16 weeks, is recommended to increase physical fitness among elderly people [46]. 

In detail, with reference to muscle strength, which represents a key variable to maintain functional fitness in older adults to prevent mobility disorders [15], the 24-week exercise intervention program was effective in improving lower body strength, whereas an increasing trend, though not significant, occurred for upper body strength. However, it should be noted that although the purpose of HST is to measure the maximum isometric strength of the hand and forearm muscles, no specific grip strength exercises were done, but they were rather focused on the proximal part of the upper limb. Furthermore, all HST values, both at T0 and T1, exceeded the 20% of relative body weight, i.e., the threshold suggested as necessary to perform daily tasks that require a firm grip (e.g., raising body weight onto a raised bus platform) [37]. This, again, may explain why we did not find significant results in handgrip strength after 24 weeks of physical activity intervention in our study group. 

Other broadly studied neuromuscular components of functional fitness in older adults are represented by body flexibility and dynamic balance, due to their relation to the ability to perform common daily tasks [41,47,48]. In particular, both right and left glenohumeral joint flexibility were significantly improved after the 24-week exercise program. Moreover, the structured exercise intervention had a positive trend on the improvement of hamstring and lower back flexibility, showing an increased number of outlier values at the end of the intervention program. However, the lowest effect observed on lower body flexibility may be explained by the fact that all the subjects enrolled in the present study already showed good performance at baseline; moreover, the sample was mainly composed of women, who, as reported in literature, show better lower joint flexibility than aged-paired men [49].

In contrast, no statistically significant differences were observed in the dynamic balance component (TUG test). Nevertheless, it should be pointed out that TUG test evaluations assessed before and after the intervention program showed that in our group the meantime for the TUG test, both at baseline (7.92 ± 1.07 s) and at T1 (7.75 ± 1.14 s), was much lower than the mean TUG time calculated in the meta-analysis conducted by Bohannon (9.4 ± 0.5 s; 95% CI) with respect to the same age category [50], suggesting that all the subjects enrolled in the present study were already well-trained in the dynamic balance fitness component. This, in turn, was in line with the evaluation of aerobic endurance, which showed that although all the subjects fell within the reference values for age [23], they all showed a significant increase in 6MWT scores at the end of the training program.

Remarkably, these data are encouraging since the physical activity intervention was well tolerated by all the subjects enrolled in the present study, as confirmed by the occurrence of no significant variations in salivary cortisol levels, which still remained within the normal range for the entire duration of the physical intervention. This, in turn, suggests that the proposed intervention program is moderate, appropriate, and not stressful for older adults. On the other hand, at the end of the training program, both a significant reduction in oxidative stress, as demonstrated by a shift of d-ROM from middle to low levels [30], as well as a substantial increase in BAP levels, which indicates a substantial improvement of the antioxidant capacity from a moderate deficiency status to an almost normal value [30,31,32], occurred. This is in line with previous literature showing that regular and adequate (age-tailored) physical activity has beneficial effects on the balance between plasma ROS production and their neutralization [51,52,53]. This represents another key point since, during aging, the body becomes more susceptible to oxidation, and intense physical activity could increase cellular ROS production, thus inducing a state of cellular damage and oxidative stress.

Beyond the final purpose of ameliorating physical health and participants’ autonomy in performing daily tasks, the EuroQoL 5-D 3-L questionnaire was administered to all study participants to self-assess their health status before and after the 24-week exercise program. The questionnaire included an EuroQoL 5-D 3-L scored descriptive system (then converted into a single index value) based on five “Dimensions” related to usual daily activities (each dimension rated on three Levels of severity), and an EQ-VAS as a “thermometer-like” measure of overall self-rated health status. The results of both evaluations showed that no significant effects on subjects’ health profiles emerged after the exercise program. 

Keeping in mind that all the participants were physically active practitioners that routinely participated in senior fitness classes, the results indicate that the performed exercise program based on functional fitness exercises kept a high level of self-perceived QoL in older adults, even after a short inactivity/detraining period, which represents an important co-factor in counteracting the psycho-emotional changes related to the unfavorable physiological changes of aging.

Finally, it is interesting to note that the present study included physically active elderly practitioners (i.e., regularly participating in standard senior fitness classes) who started the training program in January, after a period of at least four weeks of inactivity, mainly due to the suspension of the gym senior fitness class for the Christmas holidays.

Within this frame, several authors have reported significant reductions in the performance of all functional fitness components even after a relatively short detraining period (i.e., lasting at least 4/6 weeks) [54,55,56]. Since the evaluation of both physical and biomechanical parameters was not assessed after the training interruption (i.e., before starting the intervention program), we cannot rule out that the detraining period may negatively impact these variables, resulting in a significant decrease in physical performance. Thus, it is presumed that the performance of all functional fitness components observed at T0 may be lower than those before the training interruption. One of the limitations of the current study is the lack of pre-interruption data in order to evaluate whether the present training program allowed the pre-interruption condition of all functional fitness components to be regained or even to improve them. Therefore, future studies will aim to evaluate the effects of detraining periods on functional fitness and the magnitude of functional recovery following specific training programs to improve and maintain, for as long as possible, elders’ QoL and autonomy. 

Some limitations of the present study should be mentioned. The main limitation is certainly represented by the small sample size. However, it was carried out in ideal circumstances with motivated subjects who were supervised during the entire observation period by an experienced physical trainer who strictly followed each participant. This, in turn, allowed for the evaluation of the effectiveness of the exercise program intervention that was organized and conducted. Secondly, the study sample was not homogeneous, since more than 70% were women. However, as reported in the study of Silva and Menezes [57], when analyzing the association of functional capacity variables with sex, only flexibility showed a statistically significant association with sex, namely, the female group. Finally, another limitation is the absence of a control group, i.e., a group of sedentary elderly subjects.

## 5. Conclusions

Despite the above limitations, the current study has contributed to its field, since we observed that a regular, age-tailored, and multimodal program based on functional fitness exercises was able to improve, at least in part, functional fitness parameters such as physical strength and flexibility while regulating anti- and pro-oxidant activity in physically active older adults.

## Figures and Tables

**Table 1:** Reference values of d-ROMs test.

d-ROMs Test	Standard Value
Normal values	250–299 U.CARR ^1^
Borderline values	300–320
Low-level oxidative stress	321–340
Middle-level oxidative stress	341–400
High-level oxidative stress	401–500
Very high-level oxidative stress	>501

^1^ 1 U.CARR = 0.08 mg/dL of H_2_O_2_.

**Table 2:** Reference values of BAP test.

BAP Test	Standard Value
Normal values	>2200 µmol/L
Borderline values	2200–2000
Slight reduction	1999–1800
Moderate reduction	1799–1600
Strong reduction	1599–1400
Very strong reduction	<1400

**Table 3:** Demographic and anthropometric characteristics of study participants at baseline (T0) and after a 24-week training program (T1).

Measure	Baseline (T0)	Post-Intervention (T1)	*p*-Value
Age (years) *	72.8 ± 7.5	---	---
Height (m) *	1.63 ± 0.08	---	---
Weight (kg) *	68.4 ± 8.9	67.6 ± 8.7	0.10
BMI (kg/m^2^) *	25.8 ± 2.0	25.5 ± 2.2	0.13
Body fat (%) *	37.2 ± 5.7	35.7 ± 6.1	0.047

* Data are given as the mean ± SD.

## Data Availability

The data that support the findings of this study are available from the corresponding author upon reasonable request.

## References

[1] López-Otín C., Blasco M.A., Partridge L., Serrano M., Kroemer G. (2013). The hallmarks of aging. Cell.

[2] Hou Y., Dan X., Babbar M., Wei Y., Hasselbalch S.G., Croteau D.L., Bohr V.A. (2019). Ageing as a risk factor for neurodegenerative disease. Nat. Rev. Neurol..

[3] Ukraintseva S., Arbeev K., Duan M., Akushevich I., Kulminski A., Stallard E., Yashin A. (2021). Decline in biological resilience as key manifestation of aging: Potential mechanisms and role in health and longevity. Mech. Ageing Dev..

[4] Khan S.S., Singer B.D., Vaughan D.E. (2017). Molecular and physiological manifestations and measurement of aging in humans. Aging Cell..

[5] Fingerman K.L., Ng Y.T., Huo M., Birditt K.S., Charles S.T., Zarit S. (2021). Functional Limitations, Social Integration, and Daily Activities in Late Life. J. Gerontol. B Psychol. Sci. Soc. Sci..

[6] Siqueira Rodrigues B.G., Ali Cader S., Bento Torres N.V., Oliveira E.M., Martin Dantas E.H. (2010). Pilates method in personal autonomy, static balance and quality of life of elderly females. J. Bodyw. Mov. Ther..

[7] Pernambuco C.S., Rodrigues B.M., Bezerra J.C., Carrielo A., Fernandes A.D., Vale R., Dantas E. (2012). Quality of life, elderly and physical activity. Health.

[8] Moraes H., Deslandes A., Maciel-Pinheiro Pde T., Corrêa H., Laks J. (2016). Cortisol, DHEA, and depression in the elderly: The influence of physical capacity. Arq. Neuropsiquiatr..

[9] Jacomini A.M., Dias D.D., Brito J.O., da Silva R.F., Monteiro H.L., Llesuy S., De Angelis K., Amaral S.L., Zago A.S. (2017). Influence of estimated training status on anti and pro-oxidant activity, nitrite concentration, and blood pressure in middle-aged and older women. Front. Physiol..

[10] Zaleski A.L., Taylor B.A., Panza G.A., Wu Y., Pescatello L.S., Thompson P.D., Fernandez A.B. (2016). Coming of Age: Considerations in the Prescription of Exercise for Older Adults. Methodist Debakey Cardiovasc. J..

[11] Toraman N.F. (2005). Short term and long term detraining: Is there any difference between young-old and old people?. Br. J. Sports Med..

[12] Rikli R.E., Jones C.J. (1999). Development and validation of a functional fitness test for community-residing older adults. J. Aging Phys. Act..

[13] Rikli R.E., Jones C.J. (2001). Senior Fitness Test Manual.

[14] Jones C.J., Rikli R.E. (2002). Measuring functional fitness of older adults. J. Act. Aging.

[15] Stathokostas L., McDonald M.W., Little R.M., Paterson D.H. (2013). Flexibility of older adults aged 55-86 years and the influence of physical activity. J. Aging Res..

[16] Di Lorito C., Long A., Byrne A., Harwood R.H., Gladman J.R.F., Schneider S., Logan P., Bosco A., van der Wardt V. (2021). Exercise interventions for older adults: A systematic review of meta-analyses. J. Sport Health Sci..

[17] Macdonald S.H., Travers J., Shé É.N., Bailey J., Romero-Ortuno R., Keyes M., O’Shea D., Cooney M.T. (2020). Primary care interventions to address physical frailty among community-dwelling adults aged 60 years or older: A meta-analysis. PLoS ONE.

[18] Tse A.C., Wong T.W., Lee P.H. (2015). Effect of Low-intensity Exercise on Physical and Cognitive Health in Older Adults: A Systematic Review. Sports Med. Open..

[19] Lee M., Lim T., Lee J., Kim K., Yoon B. (2017). Optimal retraining time for regaining functional fitness using multicomponent training after long-term detraining in older adults. Arch. Gerontol. Geriatr..

[20] Rikli R., Jones C. (2013). Senior Fitness Test Manual.

[21] Kuzala E.A., Vargo M.C. (1992). The relationship between elbow position and grip strength. Am. J. Occup. Ther..

[22] Häger-Ross C., Rösblad B. (2002). Norms for grip strength in children aged 4–16 years. Acta Paediatr..

[23] Rikli R.E., Jones C.J. (1999). Functional Fitness Normative Scores for Community-Residing Older Adults, Ages 60–94. J. Aging Phys. Act..

[24] Heyward V.H., Gibson A. (2014). Advanced Fitness Assessment and Exercise Prescription.

[25] Podsiadlo D., Richardson S. (1991). The timed “Up & Go”: A test of basic functional mobility for frail elderly persons. J. Am. Geriatr. Soc..

[26] ATS Committee on Proficiency Standards for Clinical Pulmonary Function Laboratories (2002). ATS statement: Guidelines for the six-minute walk test. Am. J. Respir. Crit. Care Med..

[27] Pacini S., Branca J.J., Gulisano M., Levi Micheli M., Ceroti M., Ruggiero M., Morucci G. (2014). Salivary testosterone and cortisol levels to assess conditioning training program in rugby union players. Med. Sport.

[28] Trotti R., Carratelli M., Barbieri M. (2002). Performance and clinical application of a new, fast method for the detection of hydroperoxides in serum. Panminerva Med..

[29] Cornelli U., Belcaro G., Cesarone M.R., Finco A. (2013). Analysis of oxidative stress during the menstrual cycle. Reprod. Biol. Endocrinol..

[30] Mancini S., Mariani F., Sena P., Benincasa M., Roncucci L. (2017). Myeloperoxidase expression in human colonic mucosa is related to systemic oxidative balance in healthy subjects. Redox Rep..

[31] Martinovic J., Dopsaj V., Dopsaj M.J., Kotur-Stevuljevic J., Vujovic A., Stefanovic A., Nesic G. (2009). Long-term effects of oxidative stress in volleyball players. Int. J. Sports Med..

[32] Fukuda T., Kurano M., Fukumura K., Yasuda T., Iida H., Morita T., Yamamoto Y., Takano N., Komuro I., Nakajima T. (2013). Cardiac rehabilitation increases exercise capacity with a reduction of oxidative stress. Korean Circ. J..

[33] Scalone L., Cortesi P.A., Ciampichini R., Belisari A., D’Angiolella L.S., Cesana G., Mantovani L.G. (2013). Italian population-based values of EQ-5D health states. Value Health.

[34] National Research Council (US) Committee on Diet and Health (1989). Diet and Health: Implications for Reducing Chronic Disease Risk.

[35] (2010). Body Composition Data for Individuals 8 Years of Age and Older: U.S. Population, 1999–2004–2010. Vital and Health Statistics.

[36] Borrud L.G., Flegal K.M., Looker A.C., Everhart J.E., Harris T.B., Shepherd J.A. (2010). Body composition data for individuals 8 years of age and older: U.S. population, 1999–2004. Vital Health Stat. 11.

[37] Activity and Health Research (1992). Allied Dunbar National Fitness Survey: Main Findings.

[38] YMCA of the USA (2000). YMCA Fitness Testing and Assessment Manual.

[39] Monteiro A.M., Silva P., Forte P., Carvalho J. (2019). The Effects of Daily Physical Activity on Functional Fitness, Isokinetic Strength and Body Composition in Elderly Community-Dwelling Women. J. Hum. Sports Exerc..

[40] Liffiton J.A., Horton S., Baker J., Weir P.L. (2012). Successful aging: How does physical activity influence engagement with life?. Eur. Rev. Aging Phys. Act..

[41] Freiberger E., Häberle L., Spirduso W.W., Zijlstra G.A.R. (2012). Long-Term Effects of Three Multicomponent Exercise Interventions on Physical Performance and Fall-Related Psychological Outcomes in Community-Dwelling Older Adults: A Randomized Controlled Trial. J. Am. Geriatr. Soc..

[42] Monteiro A.M., Forte P., Carvalho J., Barbosa T.M., Morais J.E. (2021). Relationship between Fear of Falling and Balance Factors in Healthy Elderly Women: A Confirmatory Analysis. J. Women Aging.

[43] Monteiro A.M., Forte P., Carvalho M.J. (2020). The Effect of Three Different Training Programs in Elderly Women’s Isokinetic Strength. Motricidade.

[44] de Melo L.L., Menec V.H., Ready A.E. (2014). Relationship of functional fitness with daily steps in community-dwelling older adults. J. Geriatr. Phys. Ther..

[45] Chetty L., Ramklass S., McKune A. (2019). The effects of a structured group exercise programme on functional fitness of older persons living in old-age homes. Ageing Soc..

[46] Yang Y.-P., Lin H.C., Chen K.-M. (2019). Functional Fitness in Older Adults: A Systematic Review and Meta-analysis. Top. Geriatr. Rehabil..

[47] Carvalho M.J., Marques E., Mota J. (2009). Training and Detraining Effects on Functional Fitness after a Multicomponent Training in Older Women. Gerontology.

[48] Monteiro A.M., Bartolomeu R.F., Forte P., Carvalho J. (2019). The effects of three different types of training in functional fitness and body composition in older women. J. Sport Health Res..

[49] Lohne-Seiler H., Kolle E., Anderssen S.A., Hansen B.H. (2016). Musculoskeletal fitness and balance in older individuals (65–85 years) and its association with steps per day: A cross sectional study. BMC Geriatr..

[50] Bohannon R.W. (2006). Reference values for the timed up and go test: A descriptive meta-analysis. J. Geriatr. Phys. Ther..

[51] Rowiński R., Kozakiewicz M., Kędziora-Kornatowska K., Hübner-Woźniak E., Kędziora J. (2013). Markers of oxidative stress and erythrocyte antioxidant enzyme activity in older men and women with differing physical activity. Exp. Gerontol..

[52] Fraile-Bermúdez A.B., Kortajarena M., Zarrazquin I., Maquibar A., Yanguas J.J., Sánchez-Fernández C.E., Gil J., Irazusta A., Ruiz-Litago F. (2015). Relationship between physical activity and markers of oxidative stress in independent community-living elderly individuals. Exp. Gerontol..

[53] Gamberi T., Gorini G., Fiaschi T., Morucci G., Pratesi S., Fittipaldi L., Gulisano M., Modesti P.A., Modesti A., Magherini F. (2018). Effect of Functional Fitness on Plasma Oxidation Level in Elders: Reduction of the Plasma Oxidants and Improvement of the Antioxidant Barrier. Am. J. Sports Sci..

[54] Toraman N.F., Ayceman N. (2005). Effects of six weeks of detraining on retention of functional fitness of old people after nine weeks of multicomponent training. Br. J. Sports Med..

[55] Hallage T., Krause M.P., Haile L., Miculis C.P., Nagle E.F., Reis R.S., Da Silva S.G. (2010). The effects of 12 weeks of step aerobics training on functional fitness of elderly women. J. Strength Cond. Res..

[56] Celestrin C.P., Rocha G.Z., Stein A.M., Guadagnini D., Tadelle R.M., Saad M.J.A., Oliveira A.G. (2020). Effects of a four week detraining period on physical, metabolic, and inflammatory profiles of elderly women who regularly participate in a program of strength training. Eur. Rev. Aging Phys. Act..

[57] Silva NDA, Menezes TN De (2014). Functional capacity and its association with age and sex in an elderly population. Rev. Bras. Cineantropom. Desempenho Hum..

